# Ice Growth Inhibition in Antifreeze Polypeptide Solution by Short-Time Solution Preheating

**DOI:** 10.1371/journal.pone.0154782

**Published:** 2016-05-06

**Authors:** Naoto Nishi, Takuya Miyamoto, Tomonori Waku, Naoki Tanaka, Yoshimichi Hagiwara

**Affiliations:** 1 Department of Mechanical and System Engineering, Graduate School of Science and Technology, Kyoto Institute of Technology, Matsugasaki, Sakyo-ku, Kyoto, 606–8585, Japan; 2 Faculty of Molecular Chemistry and Engineering, Kyoto Institute of Technology, Matsugasaki, Sakyo-ku, Kyoto, 606–8585, Japan; 3 Faculty of Mechanical Engineering, Kyoto Institute of Technology, Matsugasaki, Sakyo-ku, Kyoto, 606–8585, Japan; The Ohio State University, UNITED STATES

## Abstract

The objective of this study is to enhance the inhibition of ice growth in the aqueous solution of a polypeptide, which is inspired by winter flounder antifreeze protein. We carried out measurements on unidirectional freezing of the polypeptide solution. The thickness of the solution was 0.02 mm, and the concentration of polypeptide was varied from 0 to 2 mg/mL. We captured successive microscopic images of ice/solution interfaces, and measured the interface velocity from the locations of tips of the pectinate interface in the images. We also simultaneously measured the temperature by using a small thermocouple. The ice/solution interface temperature was defined by the temperature at the tips. It was found that the interface temperature was decreased with an increasing concentration of polypeptide. To try varying the activity of the polypeptide, we preheated the polypeptide solution and cooled it before carrying out the measurements. Preheating for 1–5 hours was found to cause a further decrease in the interface temperature. Furthermore, wider regions of solution and ice with inclined interfaces in the pectinate interface structure were observed, compared with the case where the solution was not preheated. Thus, the ice growth inhibition was enhanced by this preheating. To investigate the reason for this enhancement, we measured the conformation and aggregates of polypeptide in the solution. We also measured the local concentration of polypeptide. It was found that the polypeptide aggregates became larger as a result of preheating, although the polypeptide conformation was unchanged. These large aggregates caused both adsorption to the interface and the wide regions of supercooled solution in the pectinate interface structure.

## Introduction

The inhibition of ice growth is an important issue in various fields, such as the maintenance of the quality of food texture in food preservation [1, 2]; the storage of cells, tissues and organs in hospitals [1, 3]; and cryosurgery [4]. Antifreeze protein (AFP) and antifreeze glycoprotein (AFGP) have been investigated in relation to the inhibition of ice growth [1–3]. This is because the AF(G)P solutions have the following non-colligative properties: (a) the freezing point drop is not proportional to the AF(G)P concentration, (b) the melting point is retained, (c) the osmotic pressure does not significantly increase, and (d) specific pyramidal facets are observed on the surfaces of tiny ice crystals in AFP solutions in osmometers and on the surfaces of single-crystal ice-hemispheres which are grown from the end of a brass cold finger inserted into the surface of the solution [5, 6]. It should be noted that these freezing and melting points were defined to be respectively the temperatures at which a small seed crystal started to grow and shrink in the solution in an osmometer [7, 8].

Some of these properties of AF(G)P solutions can be explained by the following hypotheses concerning the interaction between AF(G)P molecules and water molecules: (i) AF(G)P is adsorbed onto the specific facets of the ice crystals in a manner in which some hydrophilic residues on one or two sides of the AF(G)P are bound to these facets by the hydrogen bond, and (ii) some hydrophobic residues on the other sides of the AF(G)P form hydrophobic hydration shells. Furthermore, properties (a) and (b) which are mentioned above can be evaluated by a model with free energy as follows [9, 10]: The AF(G)P molecules adsorbed onto the ice surface noticeably enhance the peak (or barrier) of the excess free energy at a critical radius of interface curvature. The excess free energy is the sum of the positive interface free energy and the negative volume free energy. Thus, the adding of AF(G)P to water is promising for ice growth inhibition.

When using AF(G)P, the following three factors should be considered: (1) the cooling rate or the rate of ice growth, (2) protein denaturation, and (3) enhancement of supercooled states. Regarding factor (1), the absolute values of the cooling rate (*K*) and the rate of ice crystal growth (*R*) in the osmometers (e.g. *K* = 0.074°C/min [11] and *R* = 0.2 μm/s [8]) are much lower than those in cryosurgery (*K* = 10°C/min and *R* ≈ 30–40 μm/s [4]). To elucidate the effects of these cooling rates on the shape, temperature and growth rate of ice crystals in the solution of AF(G)P, experimental results for higher rates of ice growth, such as results for unidirectional freezing of AF(G)P solutions, are required. Thus, many experiments on unidirectional freezing have been carried out [12–16]. From the results in [13, 15, 16], in the case of AF(G)P solutions, it was found that the decrease in the temperature at the most advanced point of the serrated interface is consistent with the freezing point drop measured with osmometers, although the interface temperature depended on the interface velocity.

Regarding factor (2), as far as we know, the effect of protein denaturation on the freezing of AF(G)P solution has not been discussed in detail. Recently, Kun and Mastai [17] synthesized three polypeptides based on parts of winter flounder AFP. Using an osmometer, they measured the non-colligative freezing point drop for a solution of one of these polypeptides. Our research group expects that denaturation of the polypeptide does not occur in this case because the short helical structure of the polypeptide, which includes many hydrophobic residues, is maintained with strong hydrophobic interaction and the hydrogen bonds.

Regarding factor (3), the enhancement of supercooled states has not yet been discussed in detail except as a synergetic effect obtained from the measurements with osmometers [18, 19]. In ref. [18], the freezing point drop for the mixed solution had the following three components: (1) a colligative portion produced by the salts; (2) a portion produced by AF(G)P alone; and (3) a synergetic portion produced by the interaction of the salts and AF(G)P. It can be surmised from the results for unidirectional freezing shown in ref. [16] that the decrease in the temperature at the tip of the ice/solution interface had three similar components as follows: (1) a portion similar to the freezing point drop produced by sodium chloride or sodium permanganate, (2) a portion produced by winter flounder AFP alone, and (3) a synergetic portion produced by the interaction of these salts and AFP. However, in the case of a mixed solution of polypeptide and sodium chloride, the present authors have been unable to obtain such a clear effect, except for the case with a high concentration of the polypeptide [20]. Thus, we concluded that other stimuli or stresses on the polypeptide solution are required to enhance the supercooled states.

In the present study, we experimentally investigate the following two factors for the polypeptide: the ice morphology and interface temperature in the unidirectional freezing of polypeptide solution (related to factor (1) mentioned in the third paragraph of this section), and whether or not the morphology and temperature change as a result of preheating the polypeptide solution (related to factors (2) and (3)).

## Materials and Methods

### Apparatus

The apparatus consisted of an inverted microscope (Nikon C2+), a monochrome charge-coupled device video camera, a digital multi-meter (Yokogawa 7561) and a pulse generator. The light source was a halogen lamp. The apparatus was set up in a temperature-controlled room maintained at 8°C.

**Fig 1:**
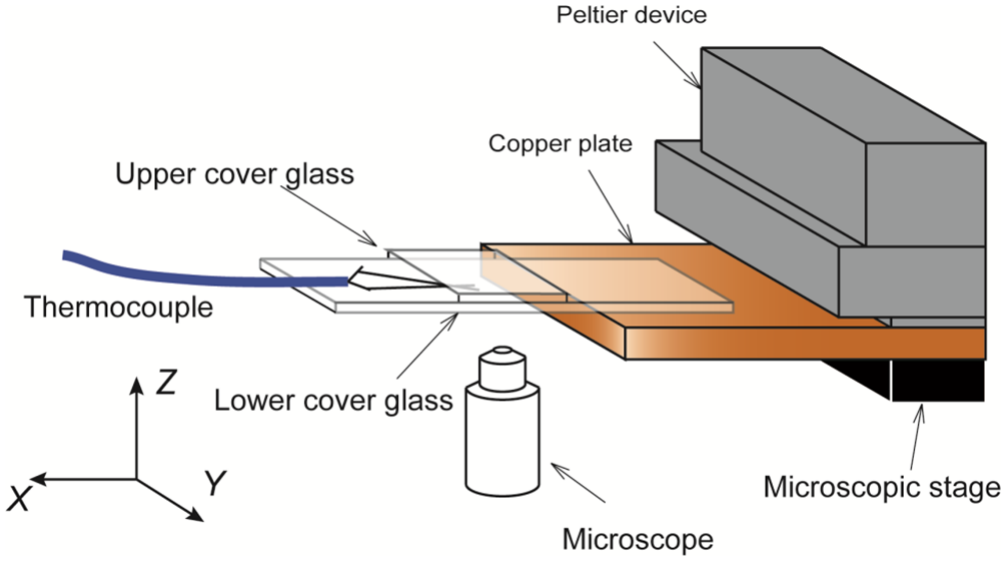
Details of cooling section. The solutions were stored in a space of 25 mm × 22 mm × 0.02 mm between parallel cover glasses. The gap between the cover glasses was realized by the presence of a screen, printed on the lower side of the upper cover glass. The transparent cover and plastic film were used to reduce the influence of air-conditioning flow on the measurements.

Fig 1 shows the details of the cooling section mounted on the bench of the microscope. A dilute aqueous solution of polypeptide or AFP was introduced into a narrow space of 11 mm^3^ (25 mm × 22 mm × 0.02 mm) between parallel cover glasses by the capillary action of the liquid. The gap of 0.02 mm between the cover glasses was created by using a screen printed on the lower side of the upper cover glass. The screen controls the growth direction of the ice crystal. The lower cover glass was in contact with the edge of the copper plate. This plate was cooled by a Peltier device with a coolant flowing through the device. The cooling rate of the device was controlled with a controller (Sensor Control Inc. Japan, FC3510). This controller automatically controlled the Peltier device from the temperature measured with a thermocouple inside the Peltier device and a predetermined temperature decrease rate. To obtain a low temperature gradient, we operated the controller of the Peltier device at its lowest temperature-drop rate of *K* = 1.0°C/min, which is 35% higher than that in the osmometers. In addition, ice formed naturally from the cooler edge of the solution layer without any seed crystals.

### Materials

#### Polypeptide

Table 1 shows the primary structure and molecular weight of the polypeptide used in this study. This primary structure is the same as part of the primary structure of winter flounder AFP mentioned below. A helical secondary structure is expected from the discussion in ref. [17], though the C-terminus of the polypeptide was amidated. We purchased the synthetic polypeptide from GenScript Inc. (Taito, Tokyo, Japan). The polypeptide was dissolved in the Milli-Q water. The concentration of polypeptide solution was 1mg/mL or 2 mg/mL.

**Table 1:** Polypeptide and Winter Flounder Antifreeze Protein.

Material	Primary structure	Molecular weight
Polypeptide	DTASDAAAAAAL	1046 Da
Winter flounder AFP	DTASDAAAAAALTAANAKAAAELTAANAAAAAAATAR	3243 Da

D, Aspartate; T, Threonine; A, Alanine; L, Leucine; N, Asparagine; K, Lysine; E, Glutamic acid; R, Arginine.

The solution of the polypeptide was preheated at a predetermined temperature for a predetermined period of time. In the case of the experiments investigating unidirectional freezing, a plastic bottle containing the sample liquid (1 mg) was installed in a thermostatic bath (Yamato Scientific Co., Ltd, Japan, BB301) in the preheating procedure. The predetermined temperature of the thermostatic bath was set to 80°C. The predetermined duration was in the range of 1–24 hours. After preheating, the sample liquid was cooled in a constant-temperature room at 8°C. The concentration of preheated polypeptide solution was the same as that for the unheated solution.

In the other experiments described in the section on measurement methods, the sample liquid (10 μL) was heated using a copper bath in the preheating procedure. The predetermined temperature of preheating was the same as that for the experiments on unidirectional freezing, while the predetermined duration of preheating was shorter than that for the experiments on unidirectional freezing.

#### Winter flounder AFP

Winter flounder AFP (HPLC6) was used as a reference. The primary structure and molecular weight of HPLC6 are shown in Table 1. The secondary structure of HPLC6 is an α-helix. We purchased synthetic HPLC6 from Life Technologies Corporation (Carlsbad CA, USA). The concentration of HPLC6 solution was the same as that for the polypeptide solution.

### Measurement methods

We measured the configuration, velocity and temperature of interfaces. The measurement methods were the same as those used in our previous study [16]. We also measured the circular dichroism, transmitted electron beam and the absorbance of ultraviolet light for the solutions.

#### Interface velocity

The direction of movement of the interface was parallel to the X axis with a margin of approximately 20° regardless of the solute and its concentration. The interface configuration also did not noticeably change during the image-capturing period. Thus, the interface velocity was defined as the distance (between the tip of the convex part of an irregular interface in one captured image and that in another captured image) divided by the time interval (between the two images). The margin of error for the interface velocity was 1.7%. The image-capturing conditions are shown in Table 2.

**Table 2:** Image-capturing Conditions.

	Temperature measurements	Fluorescence microscopy
Magnification	× 20	× 20
Area size [μm^2^]	391.8 × 298.5	391.8 × 298.5
Pixel numbers	336 × 256	336 × 256
Pixel resolution [μm^2^]	1.17 × 1.17	1.17 × 1.17
Frame rate [frame/s]	1	1
Exposure time [s]	auto	0.05 (0.25)
Depth [bit]	8	8
Binning	4	4

The value in the bracket in the right-hand column shows the exposure time in the case of the preheated solution.

#### Interface temperature

A K-type thermocouple (ANBE SMT Co. Japan, KFT-13) was used to measure the temperature at a specific part of the interface. The dimensions of the thermocouple junction were approximately 60 μm × 40 μm × 10 μm. The thermocouple was inserted into the space between the cover glasses before the space was filled with liquid. The thermocouple was connected to the multi-meter, and the output from the multi-meter was recorded on a PC. The margin of error was 0.075°C.

The interface temperature was defined as the measured temperature when the tip of the convex part of an irregular interface reached the center of the thermocouple junction. Rather than the interface temperature (*T*_*i*_), we focused on the temperature difference Δ*T* = *T*_*i*_ − *T*_0_, where *T*_0_ is the average temperature at the ice/pure-water interface. Hereafter, this difference is called the decrease in the temperature at the interface.

#### Circular dichroism

To estimate the effect of preheating on the conformation of polypeptide in solutions, we measured the ellipticity of circular polarized light through the solutions using a circular dichroism spectrophotometer (JASCO, J-720). The wavelength range of the detector of this spectrophotometer was set to 190–260 nm. The sample solution was stored in a transparent cuvette. The measured ellipticity (*θ*) was converted to the mean molar residue ellipticity (*θ*) using the following equation:

$$\vartheta =\frac{\theta M}{lc},$$ (1)

where *M* is the molecular weight of the polypeptide, *c* is the molar concentration of the polypeptide, and *l* is the inner dimension of the cuvette.

#### Absorbance of ultraviolet and visible light

We measured the absorbance of transmitted ultraviolet and visible light through the heated solutions using a spectrophotometer (Shimadzu, UV1650PC). The sample solution was stored in a quartz cuvette. The intensity of the light passing through the cuvette was measured with the detector of this spectrophotometer. The absorbance *A*(*λ*, *T*) is expressed as follows:

$$A\left(\lambda ,T\right)=-{\text{log}}_{10}\left[I_{t}\left(\lambda ,T\right)/I_{0}\left(\lambda ,T\right)\right],$$ (2)

where *λ* is the wavelength of light, *T* is the temperature, *I*_*t*_ is the intensity of transmitted light, and *I*_*0*_ is the intensity of incident light. The temperature was maintained at 20°C throughout the absorbance measurements.

#### Transmission of the electron beam

To investigate the aggregates of polypeptide in greater detail, we observed the sample solutions using a transmission electron microscope (JEOL Ltd., JEM-1200EX II). We used a 1-μL solution dried on a copper grid as the specimen. The magnification was ×12,000 or ×40,000.

#### Dynamic light scattering

We carried out measurements of dynamic light scattering (DLS) for the polypeptide solutions using a particle size analyzer (Otsuka Electronics Co., Ltd., Japan, ELSZ-1000) to measure the size of aggregates in the solutions. The polypeptide concentration and preheating period for these measurements were the same as those for the measurements of transmission of the electron beam.

#### Fluorescence microscopy

We used fluorescence microscopy to measure the local concentration of polypeptide. This method is the same as that used in our previous studies [15, 16]. The amino-base side-chain of the C-terminus of the polypeptide was tagged with fluorescein isothiocyanate (FITC) via a linker. We purchased the synthetic polypeptide with FITC from GenScript Inc. (Taito, Tokyo, Japan). Tagging with FITC increased the molecular weight of the polypeptide by 37%.

A mercury lamp was used with a band-pass optical filter as the light source. This is because FITC emits green fluorescence with a wavelength of 520 nm when it is illuminated by light with a wavelength of 495 nm. The fluorescence passed through a dichroic mirror inside the microscope, and was captured with the CCD camera. FITC is oxidized and thus the fluorescence attenuates with time just after the light emitted from the mercury lamp starts to illuminate it [14]. To reduce the effect of this attenuation on the images, we captured images during a period when the attenuation of the fluorescence was at a minimum. In ref. [16], we confirmed that the brightness due to the fluorescence is approximately proportional to the FITC concentration in the solutions of 20 μm in thickness.

Image-capturing conditions are shown in Table 2. We increased the exposure time because of the low intensity of fluorescence in the case of the preheated solution. The margin of error for the brightness measurement was 0.39%.

## Results and Discussion

### Interface morphology

**Fig 2:**
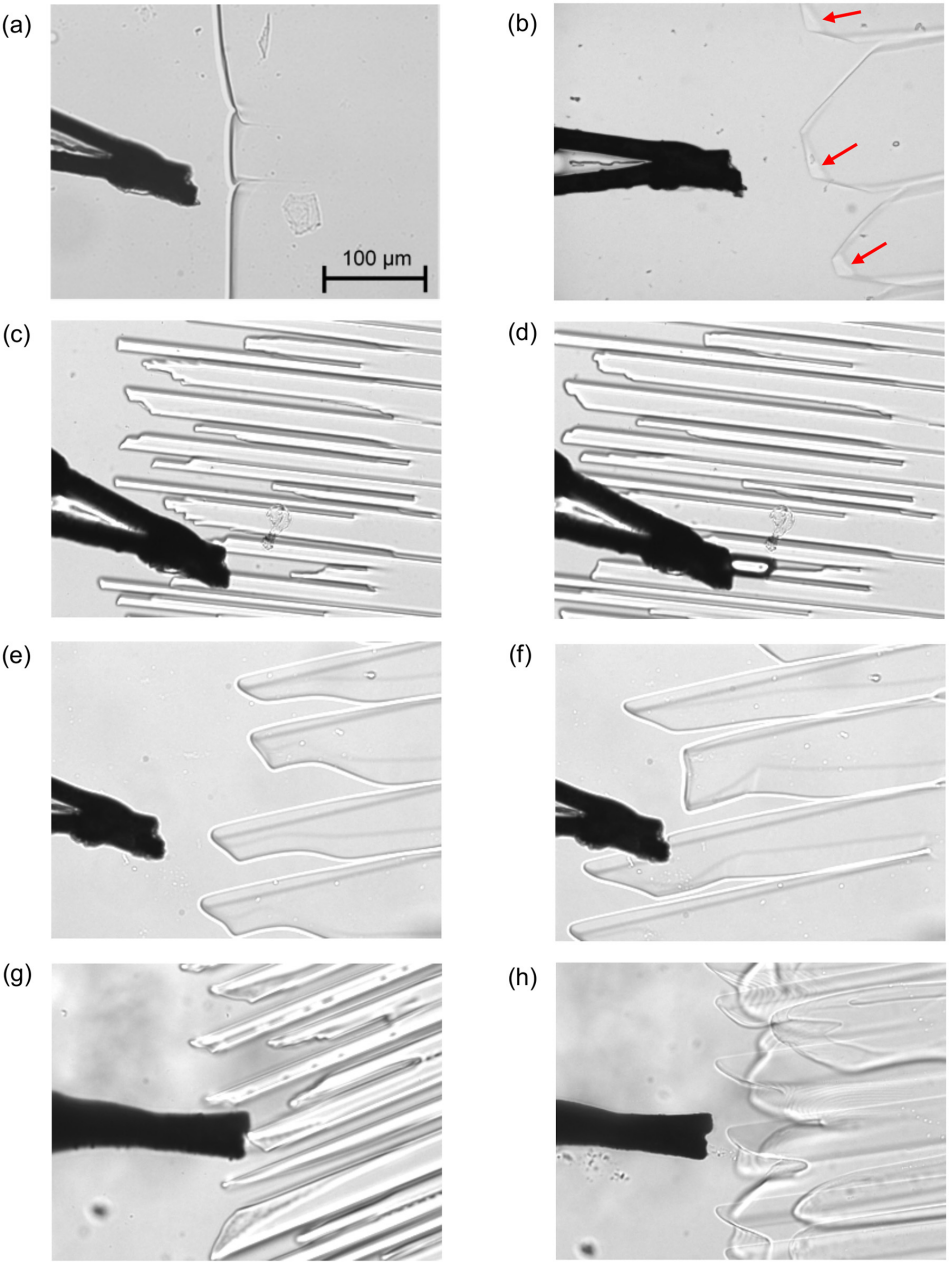
Typical images with an ice/solution interface captured with a CCD camera. The black lines on the left-hand side of each image show the element wires of the thermocouple. (a) pure water; (b) HPLC6 solution; (c) polypeptide solution (1mg/mL) at t = t_0_ s; (d) polypeptide solution (1mg/mL) at t = t_0_+5 s; (e) polypeptide solution in the case with one-hour preheating at 80°C (1mg/mL) at t = t_0_ s; (f) polypeptide solution in the case with one-hour preheating at 80°C (1mg/mL) at t = t_0_+26 s; (g) polypeptide solution in the case with 5-hour preheating at 80°C (1mg/mL); and (h) polypeptide solution in the case with 24-hour preheating at 80°C (1mg/mL).

Fig 2 shows typical shapes of the ice/liquid interface. In the case of pure water (Fig 2(a)), the interface was nearly flat and perpendicular to the ice growth direction (X direction). In the case of HPLC6 solution, however, serrated interfaces were observed (Fig 2(b)). Narrow liquid regions appeared parallel to the X axis. These regions were not observed for pure water. In addition, the thick parts of the interface were observed at the tips of the serrated interface (See the interfaces with red arrows). These parts show an inclined interface to the Z-axis.

Regarding the interface of the polypeptide solution, pectinate shapes were seen (Fig 2(c) and 2(d)). The ice regions between the liquid regions were narrower than the ice regions in the HPLC6 solution. Approximately half the liquid regions disappeared in an area 0.4 mm from the tips of the interface in the -X direction. In addition, the inclined interface was not observed for the polypeptide solution.

For the preheated solutions of polypeptide, the pitch of the teeth of the pectinate shapes in the lateral direction seemed to depend on the preheating period. The pitch was wide for 1 h preheating (Fig 2(e) and 2(f)) and 5 h preheating (Fig 2(g)), whereas the teeth were not clearly seen in the case of 24 h preheating (Fig 2(h)). Consequently, the widths of the ice regions and the solution regions were large for 1 and 5 h preheating. In addition, pairs of curves were seen along the sides of the ice ‘teeth’ regions. These curves show the inclined interfaces were not at the tips but at the sides of ice regions. Thus, the effects of preheated polypeptide on ice growth are more noticeable than the effects of non-heated polypeptide.

### Interface velocity

Table 3 shows the interface velocity. The average interface velocity for the non-preheated polypeptide solution is lower than that for pure water, but is higher than that for the HPLC6 solution with the same concentration. It should be noted that the average interface velocities for the preheated polypeptide solutions are nearly equal to or lower than that for the non-preheated HPLC6 solution, regardless of preheating periods. Thus, the preheating is effective for the inhibition of unidirectional ice growth.

**Table 3:** Interface Velocity.

	Case	Interface velocity *u*_*i*_ (μm/s)
Without preheating	Pure water	12.6 (*σ* = 2.6)
	HPLC6 solution (1mg/mL)	8.0 (*σ* = 0.5)
	Polypeptide solution (1mg/mL)	9.9 (*σ* = 2.4)
With preheating	Polypeptide solution (1mg/mL) 1 hour	7.0 (*σ* = 1.9)
	Polypeptide solution (1mg/mL) 5 hours	8.1 (*σ* = 2.8)
	Polypeptide solution (1mg/mL) 24 hours	7.2 (*σ* = 1.5)

The values in the brackets in the right-hand column show the standard deviation.

For the preheated polypeptide solutions, the dependency of the interface velocity on the interface shape was not clear. Therefore, we do not discuss here the effect of the interface velocity on the interface shapes.

### Decreases of temperature at the interface

Table 4 shows the decrease of the temperature at the tip of the ice/solution interface relative to the temperature at the ice/water interface. From this table it can be seen that the polypeptide solution without preheating shows the decrease of the temperature at the interface. The decrease is 73–82% of that of the HPLC6 solution. Thus, the polypeptide causes a supercooled state of the solution.

**Table 4:** Decrease in the Temperature at the Tip of the Ice/solution Interface Relative to the Temperature at the Ice/water Interface.

	Case	Decrease in the Temperature Δ*T* (°C)
Without preheating	HPLC6 solution (1mg/mL)	-0.074 (*σ* = 0.026)
	HPLC6 solution (2mg/mL)	-0.106 (*σ* = 0.0071)
	Polypeptide solution (1mg/mL)	-0.054 (*σ* = 0.028)
	Polypeptide solution (2mg/mL)	-0.087 (*σ* = 0.029)
With preheating	Polypeptide solution (1mg/mL) 1 hour	-0.188 (*σ* = 0.074)
	Polypeptide solution (1mg/mL) 5 hours	-0.169 (*σ* = 0.110)
	Polypeptide solution (1mg/mL) 24 hours	-0.040 (*σ* = 0.021)

The values in the brackets in the right-hand column show the standard deviation.

The decreases of the temperature at the interfaces for different preheating periods are also compared in the table. The decreases are significant when preheating for 1 h and 5 h, but not for 24 h preheating. The decreases in the interface temperatures are consistent with the pitch of the teeth of the pectinate shapes discussed in the subsection on Interface Morphology. The slight decrease in the interface temperature for 24 h preheating is possibly due to the thermal denaturation of polypeptide by the long period of heating. It can be concluded that the supercooled state of the polypeptide solution is enhanced by the stress of an appropriate preheating.

### Relationship between interface velocities and the decreases of temperature at the interface

**Fig 3:**
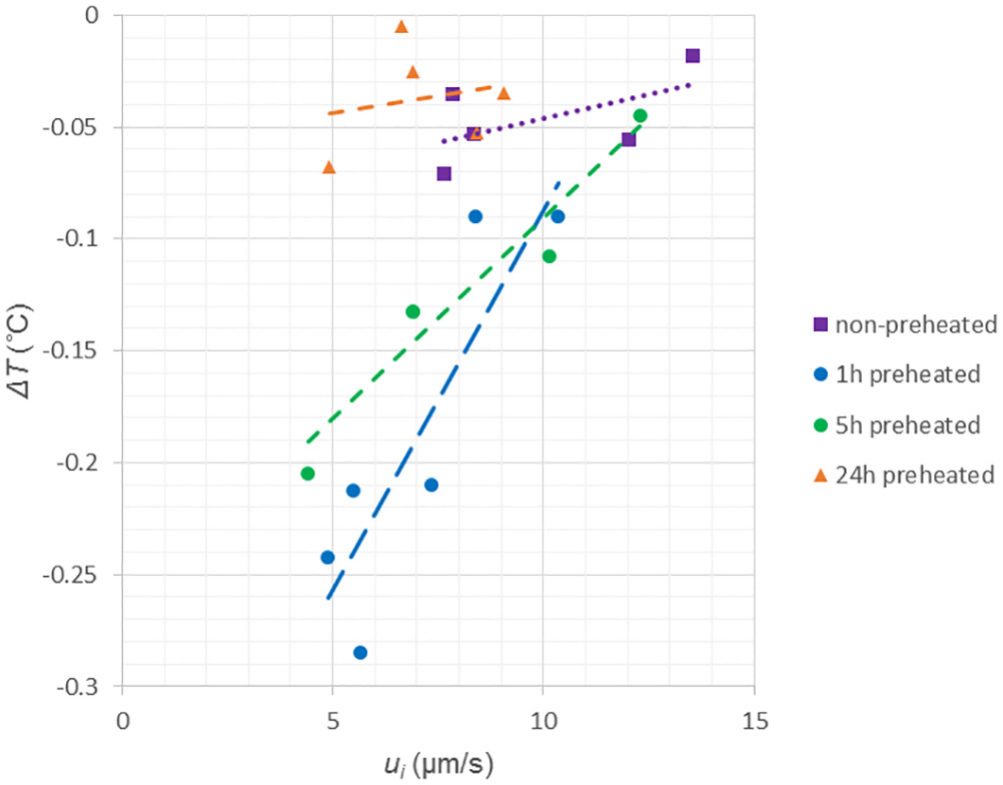
Relationship between the interface velocities and the decreases of temperature at the interface for four solutions (1mg/mL). A fitting line for the data points is also shown in each solution.

Fig 3 shows the simultaneously obtained results of the interface velocity and the decrease of temperature at the interface for several runs for each of four peptide solutions (1mg/mL). The broken lines and the dotted line in this figure are the fitting lines for the data points. The freezing point drop, mentioned in the Introduction section, can be calculated by the extrapolation of the fitting lines to the zero interface velocity. In the case of the non-preheated solution, the decrease of temperature at the interface is found to become slightly noticeable with a decrease in the interface velocity. The calculated freezing-point drop is -0.089°C. The freezing point drop can be also estimated from the thermal hysteresis (the difference between the melting point and the freezing point) obtained in ref. [17]. The estimated freezing-point drop is -0.10°C. Thus, the relationship between the interface velocities and the decreases of temperature at the interface for the non-preheated solution is consistent with the freezing point depression in the osmometers.

In the case of the solution preheated for 1 hour, the decrease of temperature at the interface becomes remarkable with a decrease in the interface velocity. The gradient of the fitting line is approximately 8 times larger than that for the non-preheated solution. The calculated freezing-point drop is -0.43°C. Similarly, in the case of the solution preheated for 5 hours, the decrease of temperature at the interface becomes remarkable with a decrease in the interface velocity. The gradient of the fitting line is approximately 4 times larger than that for the non-preheated solution and the calculated freezing-point drop is -0.27°C. Thus, the effects of polypeptides on the temperature at the interface become significant as a result of preheating for 1 hour or 5 hours, regardless of the interface velocity if it is lower than 10 μm/s.

In the cases of the solution preheated for 24 hours, on the other hand, the decreases of temperature at the interface do not become noticeable with a decrease in the interface velocity. The gradient of the fitting line is approximately 30% smaller than that for the non-preheated solution and the calculated freezing-point drop is -0.059°C. Thus, the preheating for 24 hours does not contribute to the decrease of temperature at the interface, regardless of the interface velocity.

### Polypeptide conformation

**Fig 4:**
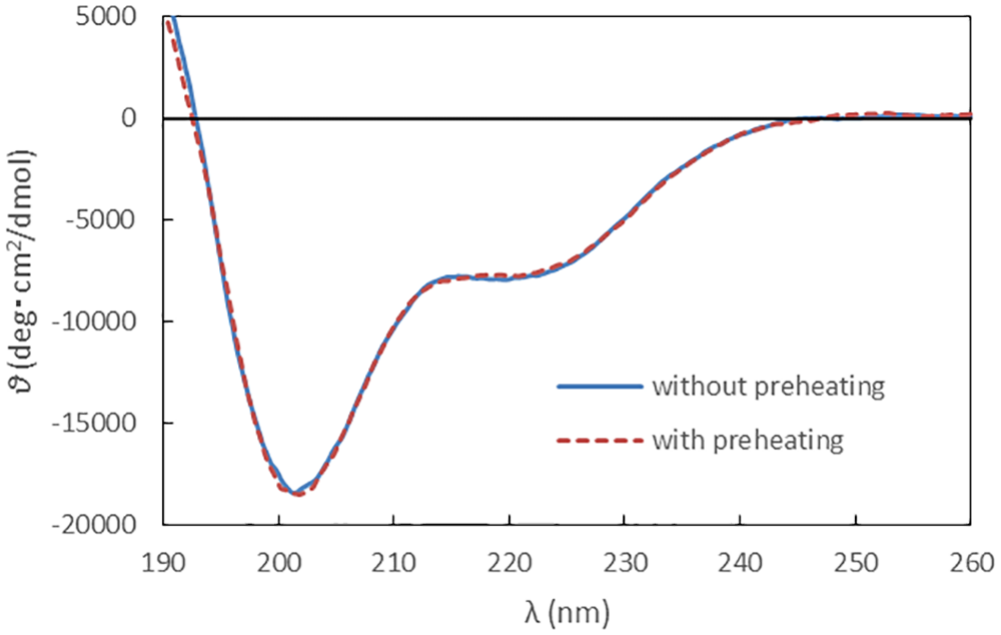
Circular dichroism spectrometry in the ultraviolet wavelength range. The polypeptide solution of 1mg/mL was preheated for one hour at 80°C.

Fig 4 shows circular dichroism spectrometry in the wavelength range of ultraviolet light (*λ*). In the non-preheated solution, the mean molar residue ellipticity has two minima at *λ* ≈ 202 and 220 nm. After preheating at 80°C for 1 h, the mean molar residue ellipticity is nearly the same as that for the non-preheated solution. Thus, it can be concluded that preheating for 1 h does not affect the polypeptide conformation in solution.

### Absorbance of light

**Fig 5:**
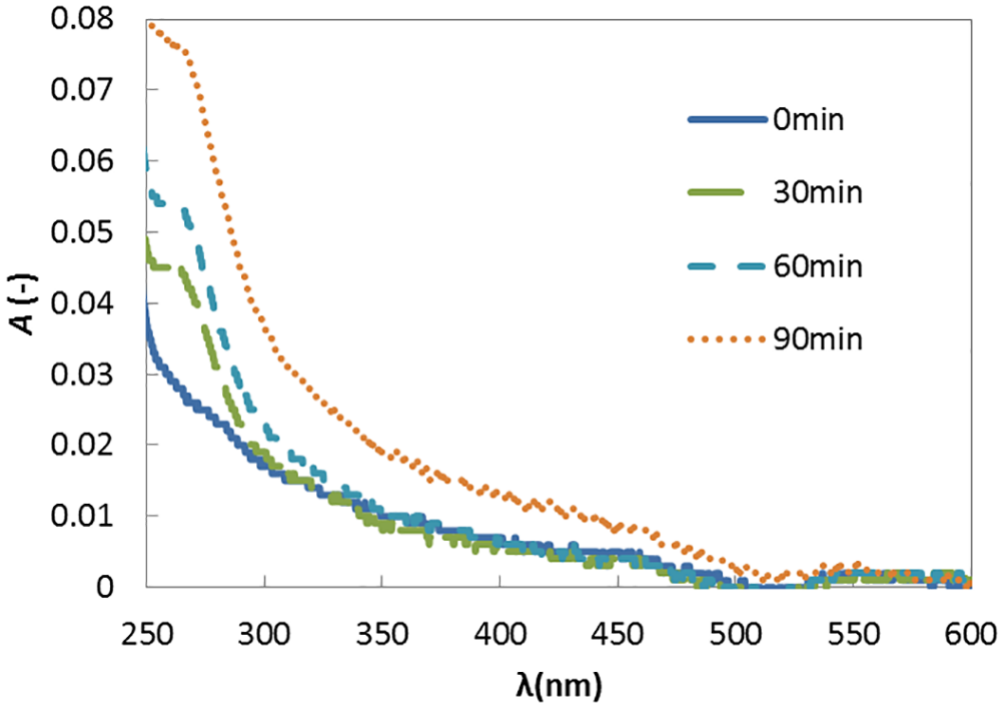
Effect of preheating period on the absorbance of ultraviolet and visible light. The polypeptide solution of 1mg/mL was preheated at 80°C.

Fig 5 shows the profiles of absorbance as a function of the wavelength in the range 250–600 nm. The absorbance is highest at the lowest wavelength and noticeably decreases for all the solutions with an increasing wavelength. The longer the preheating time, the higher the absorbance in the low wavelength range is. This shows that preheating has caused a change in the solute and that ultraviolet light in this wavelength range is more scattered by the modified solute. The formation of aggregates is most probably a modification of the polypeptide as a result of preheating. Thus, the increase in the absorbance of ultraviolet light is thought to be caused by the enhancement of aggregate formation by preheating.

### Aggregates of polypeptide

Judging from the non-colligative drop of freezing point [16] and the morphology of the interface discussed in the first part of the Results and Discussion section, the polypeptide could be adsorbed to the ice surface in the same manner as that of HPLC6. It can be surmised that the adsorption force of the polypeptide is weaker than that of HPLC6 because the number of hydrophilic residues in the polypeptide is lower than that of HPLC6. These hydrophilic residues play a key role in the adsorption. The variation of the tip shapes in the interface in Fig 2(c) shows the weak adsorption force. However, the long, narrow liquid areas in the bottom regions of the pectinate interface in Fig 2(e) and 2(f) cannot be explained by the weak adsorption force in the case with preheating.

**Fig 6:**
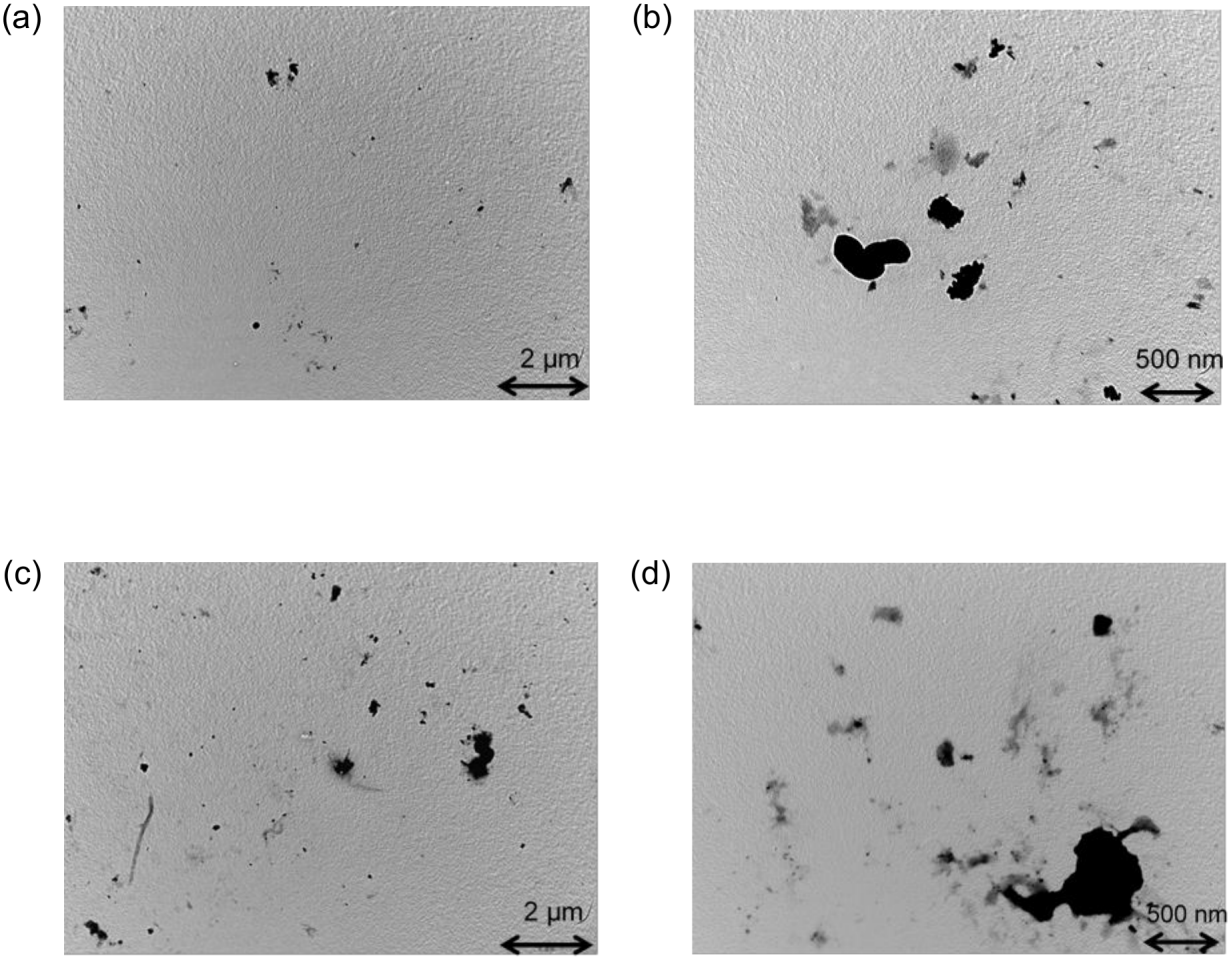
Typical images obtained with the transmission electron microscope. (a) in the case of the non-preheated solution, ×12,000; (b) in the case of the non-preheated solution, ×40,000; (c) in the case with one-hour preheating at 80°C, ×12,000; and (d) in the case with one-hour preheating at 80°C, ×40,000.

Fig 6 shows typical images obtained with the transmission electron microscope. In the non-preheated solution, small black dots are seen in Fig 6(a) and 6(b). The dimensions of these dots (*w*) seen in several images are in the range 20 < *w* < 360 nm. These dimensions are much larger than the length of the polypeptide (~ 2 nm). Thus, these dots show aggregates of polypeptides. The presence of many of these aggregates possibly contributes to the formation and maintenance of the pectinate interface.

When the solution was preheated at 80°C for 1 h, the interface became coarser, as discussed in the first part of the Results and Discussion section. It can be thought from these results that the adsorption force of the polypeptide becomes stronger as a result of the preheating of the solution. When the solution was preheated at 80°C for 1 h, black dots are also seen in Fig 6(c) and 6(d). The dimensions of these dots seen in several images are in the range 50 < *w* < 700 nm, and they are much larger than in the case of the non-preheated solution.

The DLS result for the non-preheated solution (1mg/mL) showed that the diameters of aggregates were in the range 101 < *w*_DLS_ < 263 nm, and that the average diameter was 207 nm. On the other hand, the DLS result for the solution (1mg/mL) preheated for 1 h at 80°C showed that the diameters of aggregates were in the range 175 < *w*_DLS_ < 781 nm, and that the average diameter was 523 nm. The aforementioned ranges of the dimensions of dots measured with the transmission electron microscope are consistent with these diameter ranges. Judging from these average diameters, the average volume of aggregates for the preheated solution was approximately 16 times larger than that for the non-preheated solution. Thus, preheating enhanced the formation of large aggregates. Because the width of the solution regions between two adjacent teeth of the pectinate configuration is greater and deeper with preheating than without preheating, these wider solution regions were possibly formed and maintained by the accumulation of these large aggregates.

Bouvet, Lerello and Ben [21] obtained remarkable increases in the thermal hysteresis, possibly due to the aggregates of fish AFGP in non-preheated solutions. The concentration of AFGP in their study, 20–60 mM, is much higher than the polypeptide concentration in our study. The thermal hysteresis per unit mass (or unit concentration) estimated from the thermal hysteresis activity in their study is much lower than the decreases of the interface temperature for the polypeptide solutions after preheating for 1–5 h. Therefore, the preheating of dilute solution of the polypeptide is more effective for maintaining a supercooling state than the AFGP solution in high concentration.

### Local concentration of polypeptide

**Fig 7:**
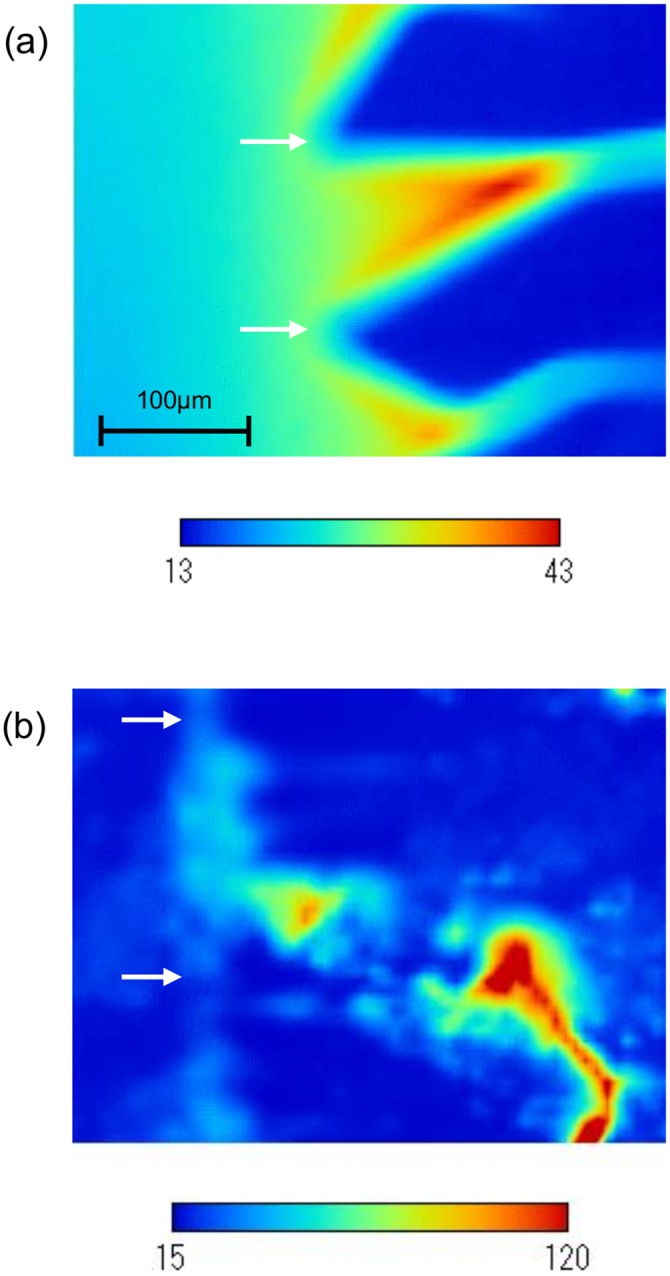
Contour maps of brightness for the polypeptide solutions. (a) non-preheated solution (1mg/mL); (b) preheated solution (1mg/mL) for 1-hour at 80°C. The arrows show the locations of tips of the interface.

Fig 7(a) and 7(b) show a typical example of the color contour maps of brightness for the non-preheated solution (1mg/mL) and that for the solution preheated for 1 hour (1mg/mL), respectively. The color scales show the brightness. The difference in the brightness between the two maps is due to the difference in the exposure time. Low brightness regions on the right-hand side of these maps show the ice regions. Although the interface shapes are not the same as those shown in Fig 2, the larger widths of the ice regions and the solution regions in the case of the preheated solution compared with those in the case of non-preheated solution are consistent with the discussion in the subsection on Interface morphology.

In the case of non-preheated solution, the brightness is nearly uniformly distributed in the direction parallel to the interface, while it decreases in the direction normal to the interface. This indicates that the concentration distribution of polypeptide did not change much along the interface and had a steep gradient in the direction normal to the interface. This is a result of the accumulation of polypeptide due to the solute exclusion effect of growing ice.

In the case of the solution preheated for 1 hour, several regions with high brightness are seen near the interface. The brightness is not uniformly distributed in the direction parallel to the interface. It decreases in the directions not only normal to the interface but also parallel to the interface. Thus, the brightness regions seem independent of each other. In addition, the regions are located close to the interface. These facts indicate that the high concentration regions of polypeptide aggregates were formed by the ice growth. It can be concluded that the high concentration regions of polypeptide aggregates interacted with part of the interface and produced the wide-pitch interface.

## Conclusions

We have carried out experiments on the gradual unidirectional freezing of the dilute solutions of polypeptide in a narrow gap between two cover glasses. This was inspired by winter flounder AFP. In addition, we measured the concentration of polypeptides, the absorbance of ultraviolet light and observed the transmitted electron beam and dynamic light scattering of the solutions being preheated. The main conclusions are as follows:

1. The decrease of the temperature at the interface for the polypeptide solution was approximately 73–82% of that of HPLC6 solution. The decrease of the temperature at the interface became significant with an increasing concentration of polypeptide. In addition, this decrease became more noticeable after preheating for 1–5 h. Thus, the supercooled state of the polypeptide solution is enhanced by the stress of an appropriate period of preheating.
2. Many small aggregates of polypeptides in the case of the non-preheated solution contributed to the formation and maintenance of a pectinate interface in this non-preheated polypeptide solution. In comparison to this, the preheating produced many large aggregates. These large aggregates contributed to the formation and maintenance of the wide regions of supercooled solution and the inclined interface. These are the main reasons for the enhancement of the supercooled states by the polypeptide aggregates.
